# ICE*Cle*SHZ29: Novel Integrative and Conjugative Element (ICE)-Carrying Tigecycline Resistance Gene *tet*(X6) in *Chryseobacterium lecithinasegens*

**DOI:** 10.3390/antibiotics14101002

**Published:** 2025-10-10

**Authors:** Xi Chen, Yifei Zhang, Chunling Jiang, Yafang Lin, Xiaohui Yao, Wansen Nie, Lin Li, Jianchao Wei, Donghua Shao, Ke Liu, Zongjie Li, Yafeng Qiu, Zhiyong Ma, Beibei Li, Lining Xia

**Affiliations:** 1 College of Veterinary Medicine, Xinjiang Agricultural University, Urmuqi 830052, China; 18241673697@163.com (X.C.); 15160870804@163.com (X.Y.); niewansen@163.com (W.N.); 2 Shanghai Veterinary Research Institute, Chinese Academy of Agricultural Sciences, Shanghai 200241, China; 13065714060@163.com (Y.L.); 15228392304@163.com (L.L.); jianchaowei@shvri.ac.cn (J.W.); shaodonghua@shvri.ac.cn (D.S.); liuke@shvri.ac.cn (K.L.); lizongjie@shvri.ac.cn (Z.L.); yafengq@shvri.ac.cn (Y.Q.); zhiyongma@shvri.ac.cn (Z.M.); 3 Shanghai Animal Disease Control Center, Shanghai 201103, China; 15300787050@163.com; 4 Shanghai Center of Agri-Products Quality and Safety, Shanghai 201708, China; jiangcl16@163.com

**Keywords:** integrative and conjugative element, antibiotic resistance, *tet*(X6), *Chryseobacterium*, swine

## Abstract

**Background/Objectives:** The global dissemination of *tet*(X) variants critically threatens tigecycline efficacy as a last-resort antibiotic. The aim of this study was to characterize a *tet*(X6)-carrying integrative and conjugative element (ICE) in a multidrug-resistant *Chryseobacterium lecithinasegens* strain, SHZ29, isolated from Shanghai, China. **Methods**: Minimum inhibitory concentrations (MICs) were determined by broth microdilution for SHZ29. Whole genomic sequencing and bioinformatic analysis were performed to depict the structure of the novel *tet*(X6)-carrying ICE. Inverse PCR and conjugation experiments were conducted to investigate the transfer ability of the ICE. **Results:** We depicted a novel *tet*(X6)-carrying ICE, named ICE*Cle*SHZ29, which is 74,906 bp in size and inserted into the 3′ end of *tRNA-Met-CAT* gene of the *C*. *lecithinasegens* strain SHZ29, with 17 bp direct repeats (5′-tcccgtcttcgctacaa-3′). This ICE possesses a 38 kb conserved backbone and four variable regions (VR1-4), with VR3 aggregating multiple resistance genes, including *tet*(X6), *tet*(X2), *erm*(F), *ere*(D), *floR*, *catB*, *sul2*, *ant*(*6*)-*I* and *bla*_OXA-1327_. NCBI database searching identified 13 additional ICEs sharing a similar backbone to ICE*Cle*SHZ29. These ICE*Cle*SHZ29-like ICEs could be classified into two types based on their distinct insertion sites: Type I, inserted at the *tRNA-Met-CAT* gene; and Type II, inserted at the *tRNA-Glu-TTC* gene. Phylogenetic analysis indicated that differences in integrases may result in differences in the insertion site among these ICEs. A circular intermediate form of ICE*Cle*SHZ29 was detected by inverse PCR. However, the conjugation experiments using *Escherichia coli* EC600 as recipients failed. **Conclusions**: To our knowledge, this study provides the first report of *tet*(X6) in *C. lecithinasegens* and characterizes its carrier, a novel ICE: ICE*Cle*SHZ29.

## 1. Introduction

The rapid dissemination of mobile antibiotic resistance genes represents a critical challenge to global public health [1]. Integrative and conjugative elements (ICEs), as a class of mobile genetic elements, play an important role in the horizontal transfer of antibiotic resistance genes [2]. They can autonomously excise from the host chromosome, transfer via conjugation in an intermediate circular form, and site-specifically insert into and replicate within a new host chromosome [3,4]. A number of clinically significant resistance genes have been found to be harbored and disseminated by ICEs, which poses serious challenges to bacterial infection treatment. For instance, the oxazolidinone resistance gene *optrA* has been documented to be harbored by the novel ICE ICE*Ssu*988S in *Streptococcus suis*, which also co-harbors the macrolide resistance gene *erm*(T) and enables horizontal transfer of multidrug-resistance among isolates [5]. The colistin resistance gene *mcr-1* was identified on the ICE ICE*Asp1* in a swine-origin *Actinobacillus* species in an Actinobacillus species of swine origin. This ICE contains a multidrug resistance region, and multiple IS*Apl1* elements mediate the formation of composite transposons to promote antibiotic resistance dissemination [6]. The carbapenem resistance gene *bla*_NDM-1_ has been identified on two novel SXT/R391 ICEs, namely ICE*Pmi*ChnXH1653 and ICE*Pvu*ChnBC22 in *Proteus mirabilis* and *P. vulgaris*, respectively. ICE*Pmi*ChnXH1653 in *P. mirabilis* harbors two tandem copies of *bla*_NDM-1_ along with 21 other antibiotic resistance genes. ICE*Pvu*ChnBC22 in *P. vulgaris* carries *bla*_NDM-1_ along with 20 other resistance genes, including *cfr* and *aac*(6′)-*Ib*-*cr*. These data indicate that ICEs serve as a key platform for the horizontal transfer of not only the *bla*_NDM-1_ gene, but also other multidrug-resistance genes [7,8].

Tigecycline is regarded as a “last-resort” antibiotic for treating multidrug-resistant Gram-negative bacterial infections [9]. However, the emergence and dissemination of plasmid-mediated mobile tigecycline resistance gene *tet*(X) variants severely compromises the clinical efficacy of tigecycline and poses a significant threat to global public health [10]. To date, at least 22 *tet*(X) variants have been reported [11]. Of them, the *tet*(X6) gene was initially identified on an SXT/R391 ICE, ICE*Pgs6Chn1*, in *Proteus* species [12]. Since then, it has been detected across a wide range of bacteria such as *Proteus*, *Acinetobacter*, *Escherichia*, and *Sphingobacterium* [13]. This gene has been documented to be present on a diverse range of ICEs. For instance, *tet*(X6) was identified on an SXT/R391-family ICE within the chromosome of *P. cibarius* isolate, where it co-occurs with a novel efflux pump gene cluster, *tnfxB3–tmexCD3–toprJ1b*, another tigecycline resistance determinant [14]. A circularizable ICE, designated ICE*EmeChn3*, that harbors the *tet*(X6) gene has been identified in *Elizabethkingia meningoseptica*. This element facilitates mobilization and recombination through IS*wz1*-mediated rearrangements, promoting the dissemination of high-level tigecycline resistance from aquaculture settings into natural ecosystems [15]. Beyond its association with ICEs, the *tet*(X6) gene has also been found to be harbored by various types of plasmids [16]. For example, the *tet*(X6) gene was identified on broad-host-range IncA/C_2_-type plasmids in animal-derived *Proteus* spp. [17]. In an *A. baumannii* isolate of chicken origin, a resistant *Pseudomonas aeruginosa* strain from poultry, the *tet*(X6) gene was located on an Inc_pRBL16_ plasmid, co-harboring *bla*_IMP-45_ and *tmexCD3–toprJ3*, which together conferred resistance to last-resort agents including tigecycline and carbapenems [18].

The genus *Chryseobacterium* are non-fermentative, oxidase-positive Gram-negative bacilli that are widely distributed in natural environments, particularly in water and soil [19]. Although *Chryseobacterium* species are generally considered low-virulence opportunistic pathogens, some members of them can cause severe clinical infections in immunocompromised patients, including respiratory tract infections, bloodstream infections, meningitis, and urinary tract infections. For instance, *C. gleum* has been reported to cause respiratory infections in neonates [19], while *C. indologenes* has been associated with urinary tract infections and other diseases [20]. Bacteria of this genus has been found to harbor various resistance genes, including several novel ones, thereby suggesting its potential role as a significant reservoir of antibiotic resistance [21]. For instance, the ICE ICE*Ci*POL15, identified in *C*. *indoltheticum*, carries the β-lactam resistance gene bla_AQU_ and has been experimentally demonstrated to transfer via conjugation [22]. ICE*CspPOL2*, identified in *C*. sp. POL2, harbors multiple antibiotic resistance genes, including a functional carbapenemase gene [23].

In the present study, we reported, for the first time, the identification of the *tet*(X6) gene in *C. lecithinasegens*. Moreover, we characterized the genetic structure and mobile ability of the *tet*(X6) carrier, a novel ICE, namely ICE*Cle*SHZ29.

## 2. Results and Discussion

### 2.1. C. lecithinasegens SHZ29 Is a Multidrug-Resistant Strain

*C. lecithinasegens* SHZ29 was isolated from a fecal sample obtained from a pig farm in Shanghai, China. MIC determination revealed that *C. lecithinasegens* SHZ29 exhibited resistance to tigecycline (MIC, 16 μg/mL), tetracycline (MIC, 256 μg/mL), ceftriaxone (MIC, 64 μg/mL), meropenem (MIC, 16 μg/mL), amikacin (MIC, 256 μg/mL), ciprofloxacin (MIC, 256 μg/mL), florfenicol (MIC, 128 μg/mL), and colistin (MIC, 256 μg/mL). To investigate the molecular mechanism of tigecycline resistance in *C. lecithinasegens* SHZ29, whole-genome sequencing was performed and analysis of the resistance gene profile showed that *C. lecithinasegens* SHZ29 harbored *tet*(X2) and *tet*(X6) genes. Both *tet*(X2) and *tet*(X6) have been demonstrated to confer resistance to tigecycline [12,13,24,25]. However, a study has shown that *tet*(X2) was not transcribed and did not contribute to tigecycline resistance in *C. indologenes* [26]. Comparative analysis revealed that the *tet*(X2) gene in *C. lecithinasegens* SHZ29 shared an identical ribosome-binding site (RBS) upstream with that of the aforementioned *C. indologenes* strain, suggesting that the *tet*(X2) gene in *C. lecithinasegens* SHZ29 is also non-functional [26]. The *tet*(X6) gene, originally identified on ICE*Pgs6Chn1* in *Proteus* spp., confers high-level tigecycline resistance across diverse bacterial species [11]. To date, several naturally occurring variants of *tet*(X6) have been reported. In *C. lecithinasegens* SHZ29, the *tet*(X6) gene exhibits four amino acid substitutions compared to the *tet*(X6) prototype [13]. Functional studies have confirmed that this variant retains the ability to confer tigecycline resistance [13]. It is therefore likely that the *tet*(X6) variant is the primary determinant of tigecycline resistance in *C. lecithinasegens* SHZ29. Besides *tet*(X2) and *tet*(X6), *C. lecithinasegens* SHZ29 harbored various other resistance genes, including macrolide resistance genes *erm*(F) and *ere*(D), the β-lactam resistance gene *bla*_OXA-1327_, phenicol resistance genes *floR* and *catB*, the sulfonamide resistance gene *sul2*, the carbapenem resistance gene *bla*_IND-14_, and the aminoglycoside resistance gene *ant*(*6*)-*I*.

### 2.2. Structure of ICECleSHZ29

**Figure 1:**
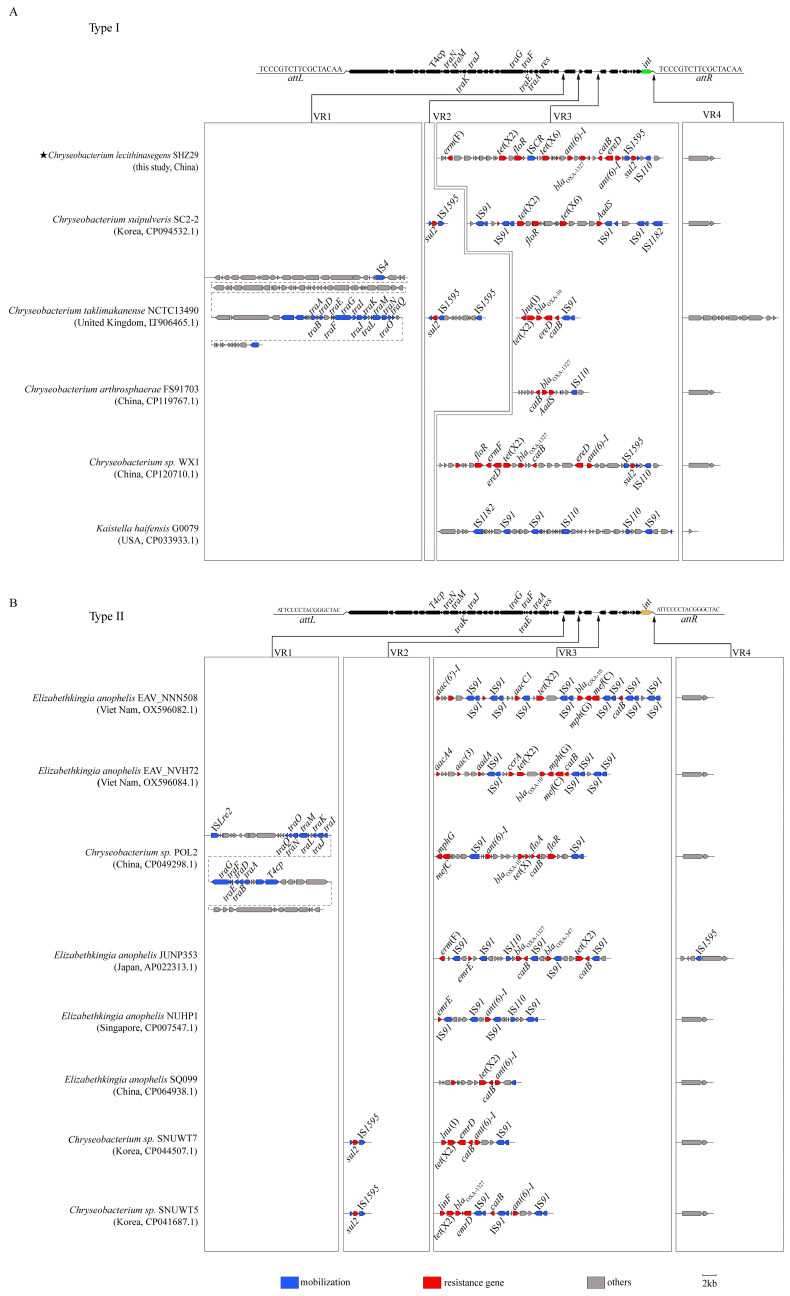
Genetic structures of ICE*Cle*SHZ29 and related ICEs. (**A**) Type I ICEs: The 6 ICEs, including ICE*Cle*SHZ29, inserted at the 3′ end of *tRNA-Met-CAT*. (**B**) Type II ICEs: The 8 ICEs inserted at the 3′ end of *tRNA-Glu-TTC*. The ICE-carrying bacteria strain names, source countries, and Genbank accession numbers are presented at the left of the pictures. The orientations and relative sizes of the ORFs are indicated by the arrows. The backbones of the ICEs are shown as the black arrows and the 4 VRs (variable regions) are exhibited in the boxes.

To investigate the genetic environment of *tet*(X6) in *C. lecithinasegens* SHZ29, the contig harboring *tet*(X6) and its flanking contigs were assembled via PCR gap filling. A novel integrative and conjugative element (ICE), designated ICE*Cle*SHZ29, was identified. ICE*Cle*SHZ29 is 74,906 bp in size with a G/C content of 38%. The element is flanked by a 17 bp perfect direct repeat (5′-tcccgtcttcgctacaa-3′) at both ends and is inserted into the 3′ end of the *tRNA-Met-CAT* gene. RAST annotation identified 90 open reading frames (ORFs) within ICE*Cle*SHZ29. Among these, some ORFs were predicted to encode core functional components of ICE, including those involved in site-specific recombination and integration (integrase and resolvase) and conjugative transfer (TraAEGJKMN and T4CP). Furthermore, ICE*Cle*SHZ29 harbored a gene cluster consisting of nine antibiotic resistance genes, including *tet*(X2), *tet*(X6), *erm*(F), *ere*(D), *floR*, *catB*, *sul2*, *ant*(*6*)-*I* (two copies), and *bla*_OXA-1327_. Within this cluster, three intact insertion sequences (IS*110*, IS*1595*, and IS*CR*), one truncated IS*1595* element, and several other ORFs encoding hypothetical proteins are interspersed among the aforementioned resistance genes (Figure 1A).

### 2.3. Phylogentic Analysis of ICECleSHZ29 

BLAST analysis of the ICE*Cle*SHZ29 revealed high homology with ICEs from multiple genera within the *Weeksellaceae* family, including *Elizabethkingia*, *Chryseobacterium*, and *Kaistella*. This family of ICEs shares a conserved backbone of about 38 kb and contains four variable regions (VR1-VR4) (Figure 1). In VR1, The ICEs from *C. taklimakanense* NCTC13490 (GenBank accession no: LT906465) and *C.* sp. POL2 (GenBank accession no: CP049298) contained several conjugative transfer-related proteins. For VR2, the ICEs from *C. suipulveris* SC2-2 (GenBank accession no: CP094532.1), *C. taklimakanense* NCTC13490, C. sp. SNU WT5 (GenBank accession no: CP041687.1), and *C.* sp. SNU WT7 (GenBank accession no: CP064938.1) carried the sulfonamide resistance gene *sul2*, flanked by IS*1595*. The VR3 region serves as an aggregation hub for antibiotic resistance genes (Figure 1). For instance, the ICE derived from *C. suipulveris* SC2-2 harbored the *tet*(X6) gene, while ICEs from *C. taklimakanense* NCTC13490, *E. anophelis* EAV_NNN508 (GenBank accession no: OX596082.1), *E. anophelis* EAV_NVH72 (GenBank accession no: OX596084.1), *C.* sp. POL2, and *C.* sp. WX1 (GenBank accession no: CP120710.1) encoded carbapenem resistance genes, such as *bla*_OXA-10_ or *bla*_OXA-347_. Moreover, similar to ICE*Cle*SHZ29, the VR3 region of these ICEs harbored diverse IS elements and intact or truncated transposase genes interspersed among these antibiotic resistance genes. The abundance of transfer-associated elements may facilitate the accumulation of antibiotic resistance genes, thereby promoting the formation of resistance gene clusters within the VR3 regions of these ICEs [27,28]. The contents of VR4 exhibit a high degree of diversity and primarily encode hypothetical proteins of unknown function.

Comparative analysis of these ICE*Cle*SHZ29-like ICEs revealed that they shared a conserved structural backbone. However, further bioinformatic analysis demonstrated that these ICEs could be classified into two distinct types based on their distinct insertion sites, designated as Type I and Type II (Figure 1). Type I ICEs insert at the 3′ end of the *tRNA-Met-CAT* gene, forming a perfect 17 bp direct repeat (5′-tcccgtcttcgctacaa-3′) at both insertion junctions (Figure 1A). By contrast, Type II ICEs insert at the 3′ end of the *tRNA-Glu-TTC* gene, generating a perfect 17 bp direct repeat (5′-attcccctacgggctac-3′) at both insertion junctions (Figure 1B).

**Figure 2:**
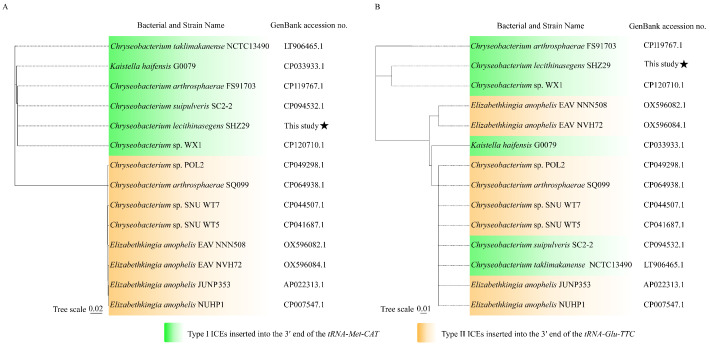
Phylogenetic trees of the integrases and relaxases of the two types of ICEs. (**A**) Integrases. (**B**) Relaxases. The ICE-carrying bacteria strain names and Genbank accession numbers are shown on the tops of the trees. The tree was constructed by a neighbor-joining method using MEGA11. Bootstrap values were calculated with 1000 replications. The ICEs inserted at the 3′ end of *tRNA-Met-CAT* and *tRNA-Glu-TTC* are marked with green and yellow, respectively.

Integrases and relaxases play critical roles in the excision and integration processes during ICE transfer [29]. Integrases, as core components of the ICE recombination module, mediate ICE integration into host chromosomes and, upon activation, cooperate with recombination directionality factors (RDFs) for ICE excision. Relaxases, central to the conjugation module, recognize the origin of transfer (oriT), cleave ICE DNA to generate single-stranded intermediates, and further facilitate their horizontal transfer via the type IV secretion system (T4SS) [30]. To investigate the insertion site divergence within ICE*Cle*SHZ29-like ICEs, we extracted the amino acid sequences of integrase and relaxase from the 14 ICEs and performed multiple sequence alignment and phylogenetic analysis. Sequence comparison revealed 4–5 amino acid differences among Type I integrases, whereas Type II integrases showed 100% amino acid identity. Substantial divergence (>100 amino acid differences) was found between Type I and Type II integrases, resulting in their clustering within distinct phylogenetic clades (Figure 2A and Supplementary Figure S1). Unlike the integrases, the relaxases of Type I and Type II ICEs did not exhibit distinct phylogenetic clustering by type. Instead, they were distributed across three divergent clades, displaying an interspersed distribution pattern (Figure 2B and Supplementary Figure S2). Previous studies have demonstrated that integrases of different types, as well as variants of different subtypes within the same family, can lead to variations in the insertion sites of ICE [31,32,33]. Taken together, the distinct insertion sites of Type I and Type II ICEs may be attributed to differences in integrases, despite sharing a conserved backbone.

**Figure 3:**
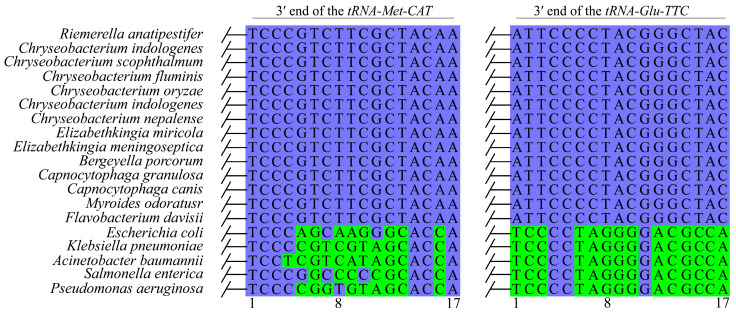
Comparison of the 3′ end sequences of the *tRNA-Met-CAT* and *tRNA-Glu-TTC* of different bacteria species. Blue and green indicate conserved and variable sequences, respectively.

To investigate the potential bacterial host range of ICE*Cle*SHZ29-like ICEs, we first performed BLAST analysis of the 3′ ends of *tRNA-Met-CAT* and *tRNA-Glu-TTC* genes, which are the attachment sites (*attB* sites) of this ICE family. These two *attB* sequences perfectly matched the 3′ ends of *tRNA-Met-CAT* and *tRNA-Glu-TTC* genes in bacterial species from the families *Flavobacteriaceae* and *Weeksellaceae*, but exhibited certain discrepancies with the 3′ ends of these two tRNA genes in other bacterial species. We then conducted a sequence comparison of the *attB* sites across diverse bacterial species (Figure 3). Representative species of the families of *Flavobacteriaceae* and *Weeksellaceae*, as well as several clinically significant Gram-negative pathogenic bacteria, including *E. coli*, *Klebsiella pneumonia*, *A*. *baumannii*, *Salmonella enterica*, and *P. aeruginosa*, were included in the analysis. Comparative analysis revealed fully conserved 3′-end sequences in *tRNA-Met-CAT* and *tRNA-Glu-TTC* genes across representative species of the *Flavobacteriaceae and Weeksellaceae* families (Figure 3). In contrast, the 3′ ends of the two tRNA genes of the five pathogenic bacteria exhibited significant differences compared with those in the families of *Flavobacteriaceae* and *Weeksellaceae*. This observation indicated that the ICE*Cle*SHZ29-like ICEs only possess the capacity for transfer exclusively among bacterial species within the families *Flavobacteriaceae* and *Weeksellaceae*, which may also explain why this ICE family has thus far been exclusively identified in *Weeksellaceae* in NCBI databases.

### 2.4. Detection of Circular Intermediate Form

**Figure 4:**
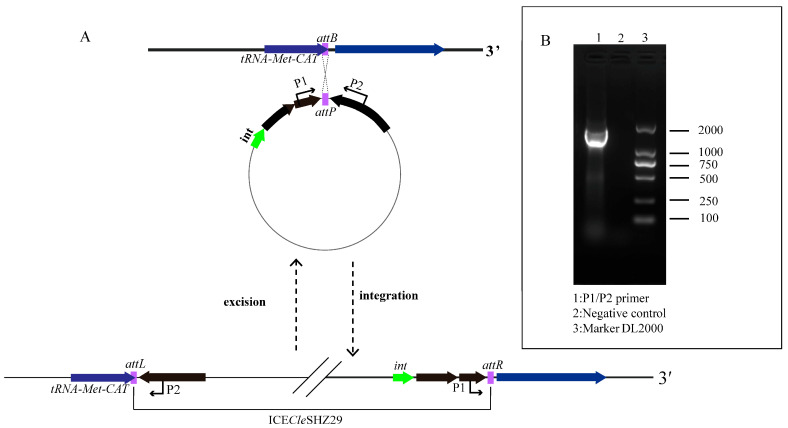
(**A**) Schematic diagram of the integration and excision of the ICE*Cle*SHZ29. (**B**) PCR detection of the circular form of ICE*Cle*SHZ29. The lane numbers and primer set are also shown.

Inverse PCR was conducted using the primer set P1/P2, with the genomic DNA of *C. lecithinasegens* SHZ29 serving as the template. The amplification yielded a positive result, indicating that ICE*Cle*SHZ29 is capable of excising itself from the chromosome and forming an intermediate circular structure (Figure 4). However, the conjugation experiment using *E. coli* EC600 as the recipient strain failed. As mentioned above, *E. coli* lacks the *attB* site of ICE*Cle*SHZ29, which may account for the failure of conjugative transfer. ICE*Cle*SHZ29 was originally isolated from *C. lecithinasegens*, a member of the *Weeksellaceae* family, and related ICEs often show host specificity for taxa closely related to their native hosts. To evaluate the transfer capacity of ICE*Cle*SHZ29, a conjugation experiment should be performed using bacterial species within the families *Flavobacteriaceae* and *Weeksellaceae*. Unfortunately, these strains were not available to us. Interestingly, a previous study demonstrated that ICE*Csp*POL2, a type II ICE*Cle*SHZ29-like ICE, fails to transfer to *E. coli* but successfully conjugates into specific *Elizabethkingia* species [23]. These findings collectively substantiate that ICE*Cle*SHZ29-like ICEs exhibit transfer competence exclusively in bacterial hosts within the *Flavobacteriaceae and Weeksellaceae* families.

## 3. Materials and Methods

### 3.1. Bacteria Strain and Antimicrobial Susceptibility Testing

During routine surveillance of tigecycline-resistant bacteria in 2024, a *C. lecithinasegens* strain designated SHZ29 was isolated from a fecal sample collected from a swine farm in Shanghai, China. Minimum inhibitory concentrations (MICs) were determined by broth microdilution according to the CLSI document M100-S30 [34,35,36]. Briefly, cation-adjusted Mueller–Hinton broth containing serially diluted test antibiotics was inoculated with the bacterial suspension at 10^5^ CFU mL^−1^. Each plate included positive controls (bacteria only) and negative controls (broth only). After incubation at 35 ± 2 °C for 16–20 h, MICs were defined as the lowest concentration of antibiotics that inhibited visible bacterial growth. To ensure the reliability of the results, we performed the MIC test with three replicates. The following antibiotics were tested: tigecycline, tetracycline, ceftriaxone, meropenem, amikacin, ciprofloxacin, florfenicol, and colistin. Given the absence of species-specific interpretive criteria for *Chryseobacterium* spp., resistance breakpoints for tetracycline, amikacin, ciprofloxacin, ceftriaxone, and meropenem were applied according to CLSI guidelines for “other non-Enterobacteriaceae” as described previously [26,37]. For tigecycline, breakpoints defined by the European Committee on Antimicrobial Susceptibility Testing (EUCAST) were utilized. Florfenicol resistance was interpreted using veterinary standards (CLSI VET08) [38]. Quality control strains *E*. *coli* ATCC 25,922 and *P*. *aeruginosa* ATCC 27,853 were included in all assays.

### 3.2. Whole Genome Sequencing and Bioinformatic Analyses

Genomic DNA of *C. lecithinasegens* SHZ29 was extracted using the TIANamp Bacterial Genomic DNA Kit (Tiangen Biotech Co., Ltd., Beijing, China). Whole genome sequencing was performed using the Illumina Hiseq 2000 platform (Majorbio, Shanghai, China), and the obtained raw data were assembled using SOAPdenovo2 [39]. Resistance genes and ICEs were identified using ResFinder [40] and ICEfinder [41], respectively. To obtain the complete sequence of the *tet*(X6)-carrying ICE, a gap-filling strategy was employed. Briefly, the contig containing *tet*(X6) was identified and used as a query in BLASTn against the NCBI nucleotide database to identify structurally related ICEs as references. All contigs from the WGS assembly were then mapped to these reference ICE sequences to identify potentially *tet*(X6)-associated contigs. Specific primer pairs were designed to span the gaps between these contigs, followed by PCR amplification (primer set P14/P16, Supplementary Table S1) and Sanger sequencing of the amplicon. The resulting amplicon sequence was then assembled with the existing tet(X6)-containing contigs to produce the closed, full-length ICE sequence. The ICE annotation was performed with the RAST server (https://rast.nmpdr.org/), followed by manual verification against the NCBI database (https://www.ncbi.nlm.nih.gov/). Sequence comparison was performed using BLAST (http://blast.ncbi.nlm.nih.gov/Blast.cgi, accessed on 24 December 2024).

### 3.3. Phylogenetic Analysis and Sequence Comparison

Phylogenetic analysis was performed for the integrases and relaxases of the ICE*Cle*SHZ29 and another 13 relevant ICEs with similar backbones. A phylogenetic tree was constructed by the neighbor-joining method using MEGA11 [42]. Bootstrap values were calculated with 1000 replications. In addition, the 3′ end nucleotide sequences of the *tRNA-Met-CAT* and *tRNA-Glu-TTC* of different bacteria species were collected and compared to explore the transfer potential of the ICE*Cle*SHZ29-like ICEs.

### 3.4. Detection of Circular Intermediates of ICECleSHZ29 and Conjugation Experiments

Circular intermediates of ICE*Cle*SHZ29 were detected by inverse PCR using primer set P1 (5′-GTGGGAACAGAAAGCGAA-3′)/P2 (5′-CTACAAACCGGAAGAAGTCG-3′). The amplicon sequence was obtained by Sanger sequencing and subjected to alignment with the *attL* and *attR* of ICE*Cle*SHZ29. To evaluate the mobility of ICE*Cle*SHZ29, conjugation experiments were conducted using rifampicin-resistant *E*. *coli* EC600 as the recipient strain. Conjugation experiments were performed as previously described with some modifications [43]. Briefly, donor and recipient strains with overnight growth were combined at a 1:1 ratio. Following centrifugation at 4000× *g* for 10 min, the cell pellet was resuspended in 100 μL of LB broth and spotted onto non-selective Tryptic Soy Agar (TSA) plates for mating. After incubation at 37 °C for 6 h, transconjugants were isolated on LB agar plates containing rifampicin (100 mg/L) and tigecycline (2 μg/mL).

### 3.5. Nucleotide Sequence Accession Number

The 74906 bp nucleotide sequence of ICE*Cle*SHZ29 has been deposited in the GenBank database and assigned the accession No. PRJNA1264973.

## 4. Conclusions

In conclusion, we identified a novel *tet*(X6)-carrying ICE, ICE*Cle*SHZ29, in a tigecycline-resistant *C. lecithinasegens* isolate of swine origin. Further studies are warranted to investigate the presence and prevalence of *tet*(X6) and ICE*Cle*SHZ29-like elements in *Chryseobacterium* species, as well as more broadly in the families *Flavobacteriaceae* and *Weeksellaceae*.

## Data Availability

The data for ICE*Cle*SHZ29 in this study can be queried in NCBI BioProject with the accession number PRJNA1264973.

## Supplementary Materials

The following supporting information can be downloaded at https://www.mdpi.com/article/10.3390/antibiotics14101002/s1: Supplementary Table S1: Primers used for PCR Assay. Supplementary Figure S1: BLAST alignment of integrase amino acid sequences from ICE*Cle*SHZ29-like integrative conjugative elements. Supplementary Figure S2: BLAST alignment of relaxases amino acid sequences from ICE*Cle*SHZ29-like integrative conjugative elements.
