# Supplementary material for: ICECleSHZ29: Novel Integrative and Conjugative Element (ICE)-Carrying Tigecycline Resistance Gene tet(X6) in Chryseobacterium lecithinasegens
Source: Antibiotics (Basel). 2025 Oct 10;14(10):1002. doi: 10.3390/antibiotics14101002 (PMC12561888; doi:10.3390/antibiotics14101002)
Supplement: Supplementary file 1 [file antibiotics-14-01002-s001.zip › Figures in PDF format/Figure 2..pdf]

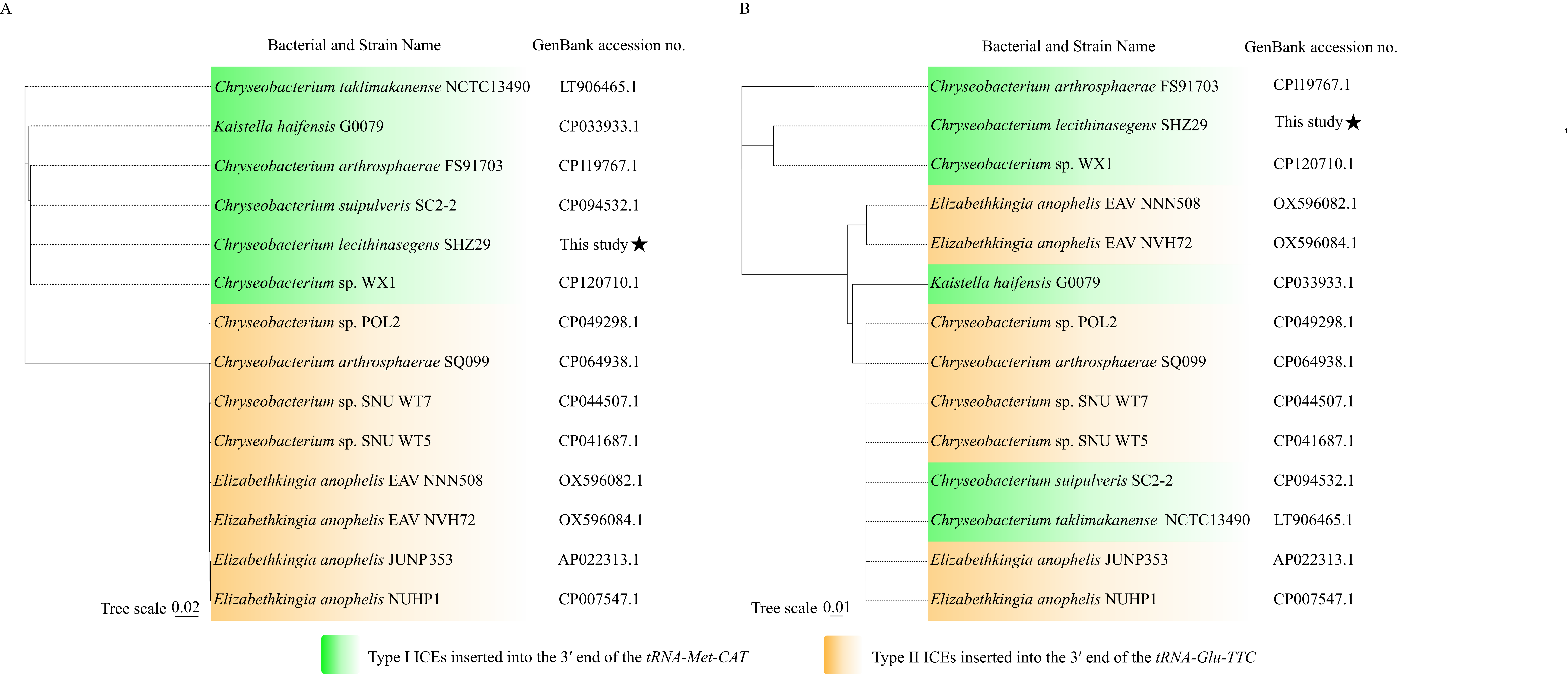
A
Bacterial and Strain Name
GenBank accession no.
Chryseobacterium taklimakanense NCTC13490 LT906465.1
Kaistella haifensis G0079 CP033933.1
Chryseobacterium arthrosphaerae FS91703 CP119767.1
Chryseobacterium suipulveris SC2-2 CP094532.1
Chryseobacterium lecithinasegens SHZ29 This study ★
Chryseobacterium sp. WX1 CP120710.1
Chryseobacterium sp. POL2 CP049298.1
Chryseobacterium arthrosphaerae SQ099 CP064938.1
Chryseobacterium sp. SNU WT7 CP044507.1
Chryseobacterium sp. SNU WT5 CP041687.1
Elizabethkingia anophelis EAV NNN508 OX596082.1
Elizabethkingia anophelis EAV NVH72 OX596084.1
Elizabethkingia anophelis JUNP353 AP022313.1
Elizabethkingia anophelis NUHP1 CP007547.1
Tree scale 0.02
B
Bacterial and Strain Name
GenBank accession no.
Chryseobacterium arthrosphaerae FS91703 CP119767.1
Chryseobacterium lecithinasegens SHZ29 This study ★
Chryseobacterium sp. WX1 CP120710.1
Elizabethkingia anophelis EAV NNN508 OX596082.1
Elizabethkingia anophelis EAV NVH72 OX596084.1
Kaistella haifensis G0079 CP033933.1
Chryseobacterium sp. POL2 CP049298.1
Chryseobacterium arthrosphaerae SQ099 CP064938.1
Chryseobacterium sp. SNU WT7 CP044507.1
Chryseobacterium sp. SNU WT5 CP041687.1
Chryseobacterium suipulveris SC2-2 CP094532.1
Chryseobacterium taklimakanense NCTC13490 LT906465.1
Elizabethkingia anophelis JUNP353 AP022313.1
Elizabethkingia anophelis NUHP1 CP007547.1
Tree scale 0.01
Type I ICEs inserted into the 3′ end of the tRNA-Met-CAT
Type II ICEs inserted into the 3′ end of the tRNA-Glu-TTC
