# Supplementary material for: ICECleSHZ29: Novel Integrative and Conjugative Element (ICE)-Carrying Tigecycline Resistance Gene tet(X6) in Chryseobacterium lecithinasegens
Source: Antibiotics (Basel). 2025 Oct 10;14(10):1002. doi: 10.3390/antibiotics14101002 (PMC12561888; doi:10.3390/antibiotics14101002)
Supplement: Supplementary file 1 [file antibiotics-14-01002-s001.zip › Figures in PDF format/Figure 3.pdf]

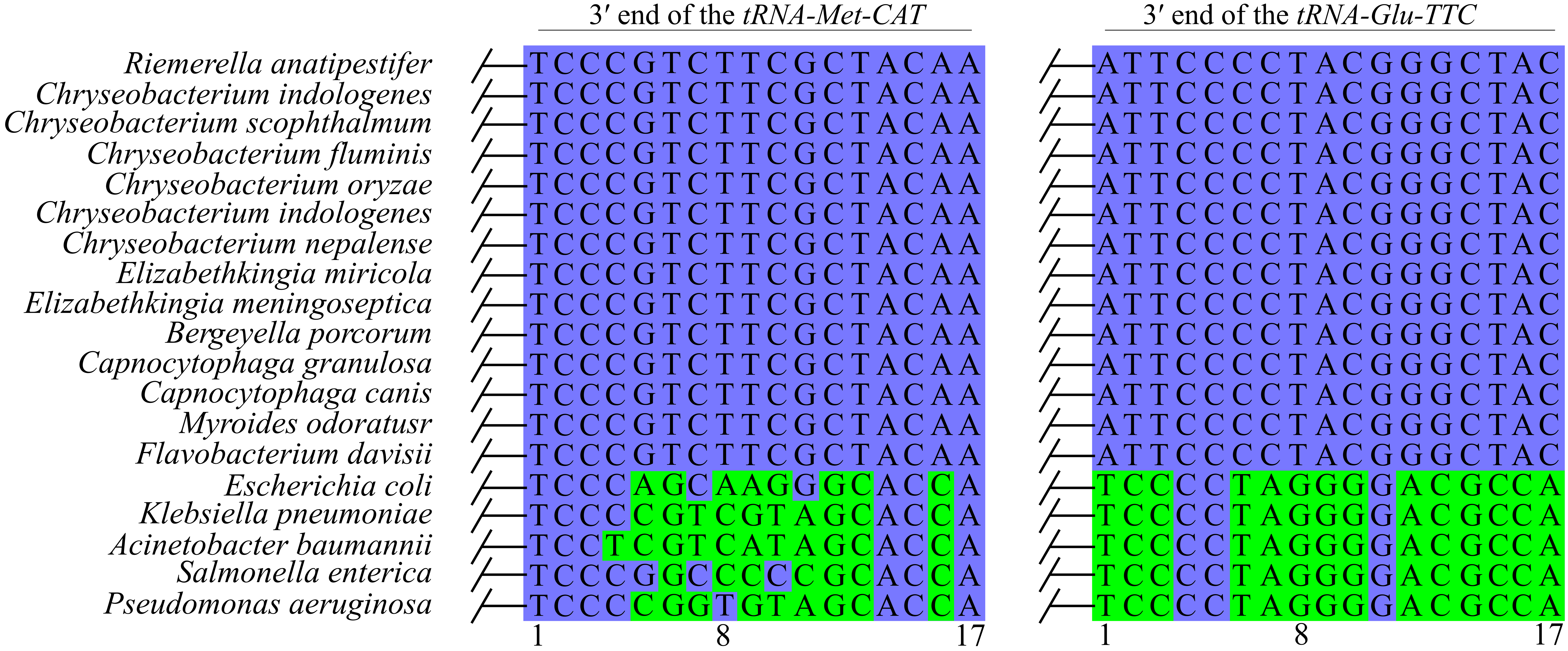

3′ end of the tRNA-Met-CAT
3′ end of the tRNA-Glu-TTC
Riemerella anatipestifer TCCCGTCTTCGCTACAA ATTCCCCTACGGGCTAC
Chryseobacterium indologenes TCCCGTCTTCGCTACAA ATTCCCCTACGGGCTAC
Chryseobacterium scophthalmum TCCCGTCTTCGCTACAA ATTCCCCTACGGGCTAC
Chryseobacterium fluminis TCCCGTCTTCGCTACAA ATTCCCCTACGGGCTAC
Chryseobacterium oryzae TCCCGTCTTCGCTACAA ATTCCCCTACGGGCTAC
Chryseobacterium indologenes TCCCGTCTTCGCTACAA ATTCCCCTACGGGCTAC
Chryseobacterium nepalense TCCCGTCTTCGCTACAA ATTCCCCTACGGGCTAC
Elizabethkingia miricola TCCCGTCTTCGCTACAA ATTCCCCTACGGGCTAC
Elizabethkingia meningoseptica TCCCGTCTTCGCTACAA ATTCCCCTACGGGCTAC
Bergeyella porcorum TCCCGTCTTCGCTACAA ATTCCCCTACGGGCTAC
Capnocytophaga granulosa TCCCGTCTTCGCTACAA ATTCCCCTACGGGCTAC
Capnocytophaga canis TCCCGTCTTCGCTACAA ATTCCCCTACGGGCTAC
Myroides odoratusr TCCCGTCTTCGCTACAA ATTCCCCTACGGGCTAC
Flavobacterium davisii TCCCGTCTTCGCTACAA ATTCCCCTACGGGCTAC
Escherichia coli TCCCAGCAAGGGCACCA TCCCCTAGGGGACGCCA
Klebsiella pneumoniae TCCCCGTCGTAGCACCA TCCCCTAGGGGACGCCA
Acinetobacter baumannii TCCTCGTCATAGCACCA TCCCCTAGGGGACGCCA
Salmonella enterica TCCCGGCCCCCGCACCA TCCCCTAGGGGACGCCA
Pseudomonas aeruginosa TCCCCGGTGTAGCACCA TCCCCTAGGGGACGCCA
1
8
17
1
8
17
