# Supplementary material for: ICECleSHZ29: Novel Integrative and Conjugative Element (ICE)-Carrying Tigecycline Resistance Gene tet(X6) in Chryseobacterium lecithinasegens
Source: Antibiotics (Basel). 2025 Oct 10;14(10):1002. doi: 10.3390/antibiotics14101002 (PMC12561888; doi:10.3390/antibiotics14101002)
Supplement: Supplementary file 1 [file antibiotics-14-01002-s001.zip › Figures in PDF format/Figure Legend.pptx]

## Slide 1
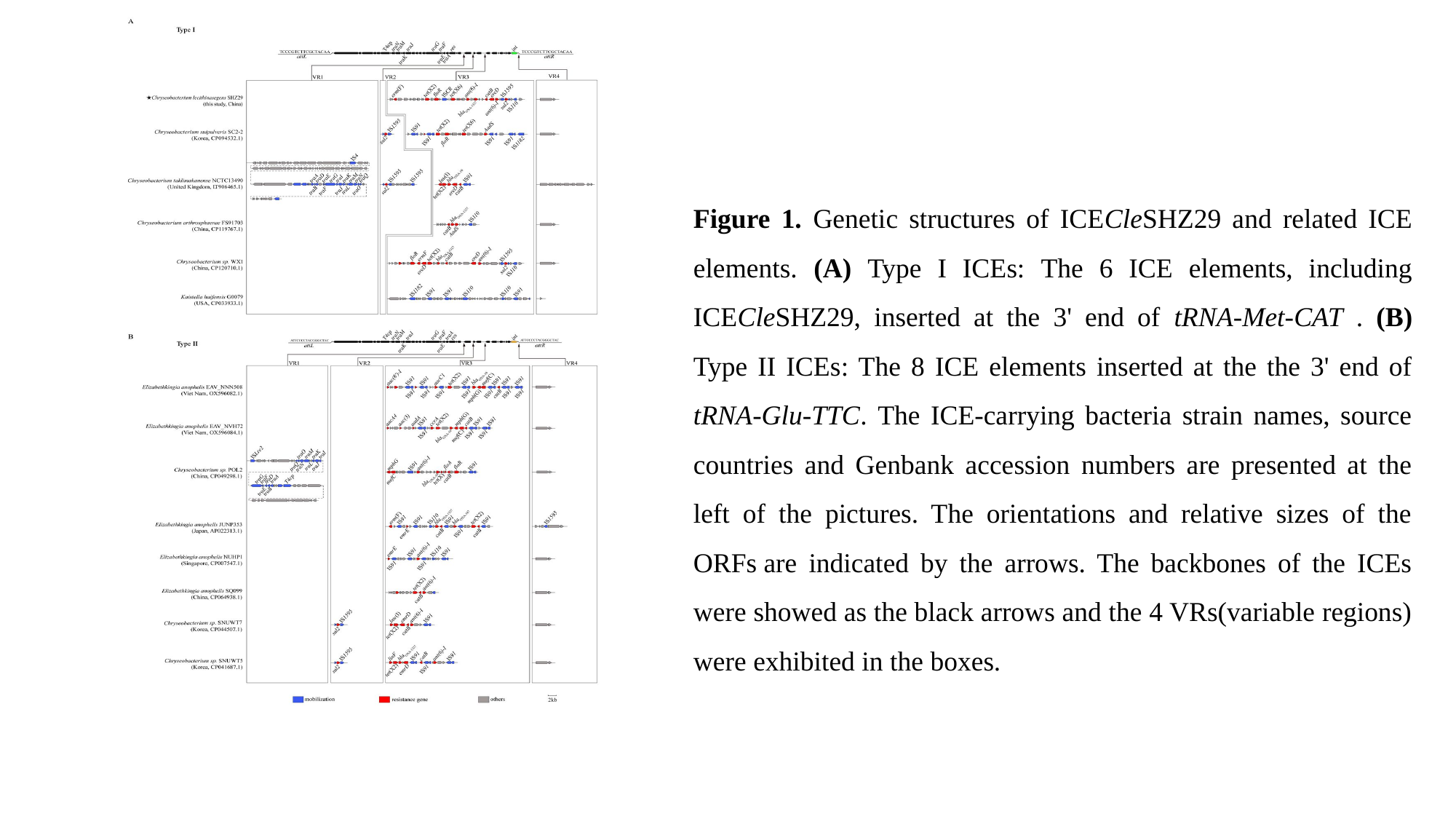

| |
| --- |
Figure 1. Genetic structures of ICECleSHZ29 and related ICE elements. (A) Type I ICEs: The 6 ICE elements, including ICECleSHZ29, inserted at the 3' end of tRNA-Met-CAT . (B) Type II ICEs: The 8 ICE elements inserted at the the 3' end of tRNA-Glu-TTC. The ICE-carrying bacteria strain names, source countries and Genbank accession numbers are presented at the left of the pictures. The orientations and relative sizes of the ORFs are indicated by the arrows. The backbones of the ICEs were showed as the black arrows and the 4 VRs(variable regions) were exhibited in the boxes.

## Slide 2
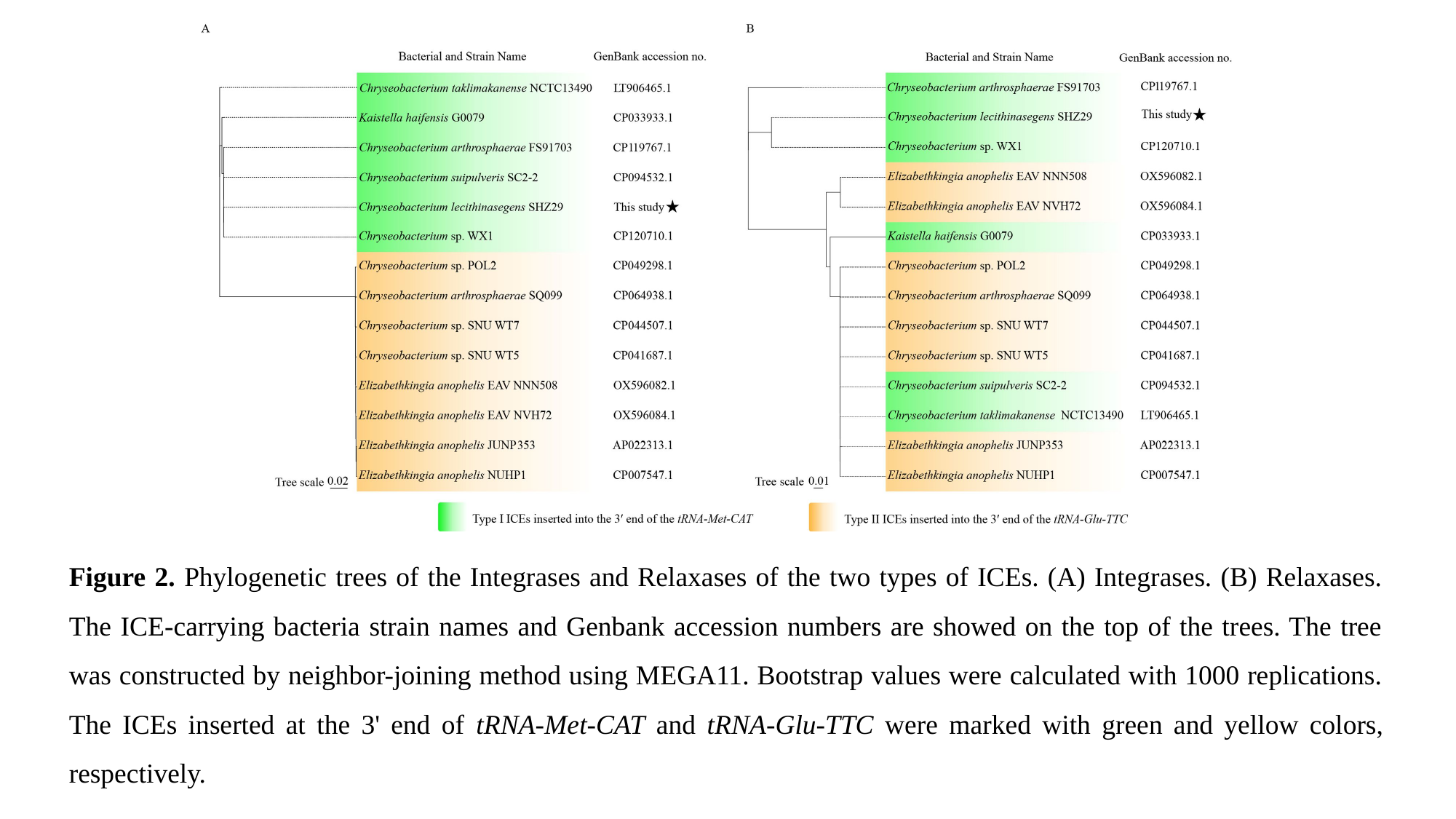

| |
| --- |
Figure 2. Phylogenetic trees of the Integrases and Relaxases of the two types of ICEs. (A) Integrases. (B) Relaxases. The ICE-carrying bacteria strain names and Genbank accession numbers are showed on the top of the trees. The tree was constructed by neighbor-joining method using MEGA11. Bootstrap values were calculated with 1000 replications. The ICEs inserted at the 3' end of tRNA-Met-CAT and tRNA-Glu-TTC were marked with green and yellow colors, respectively.

## Slide 3
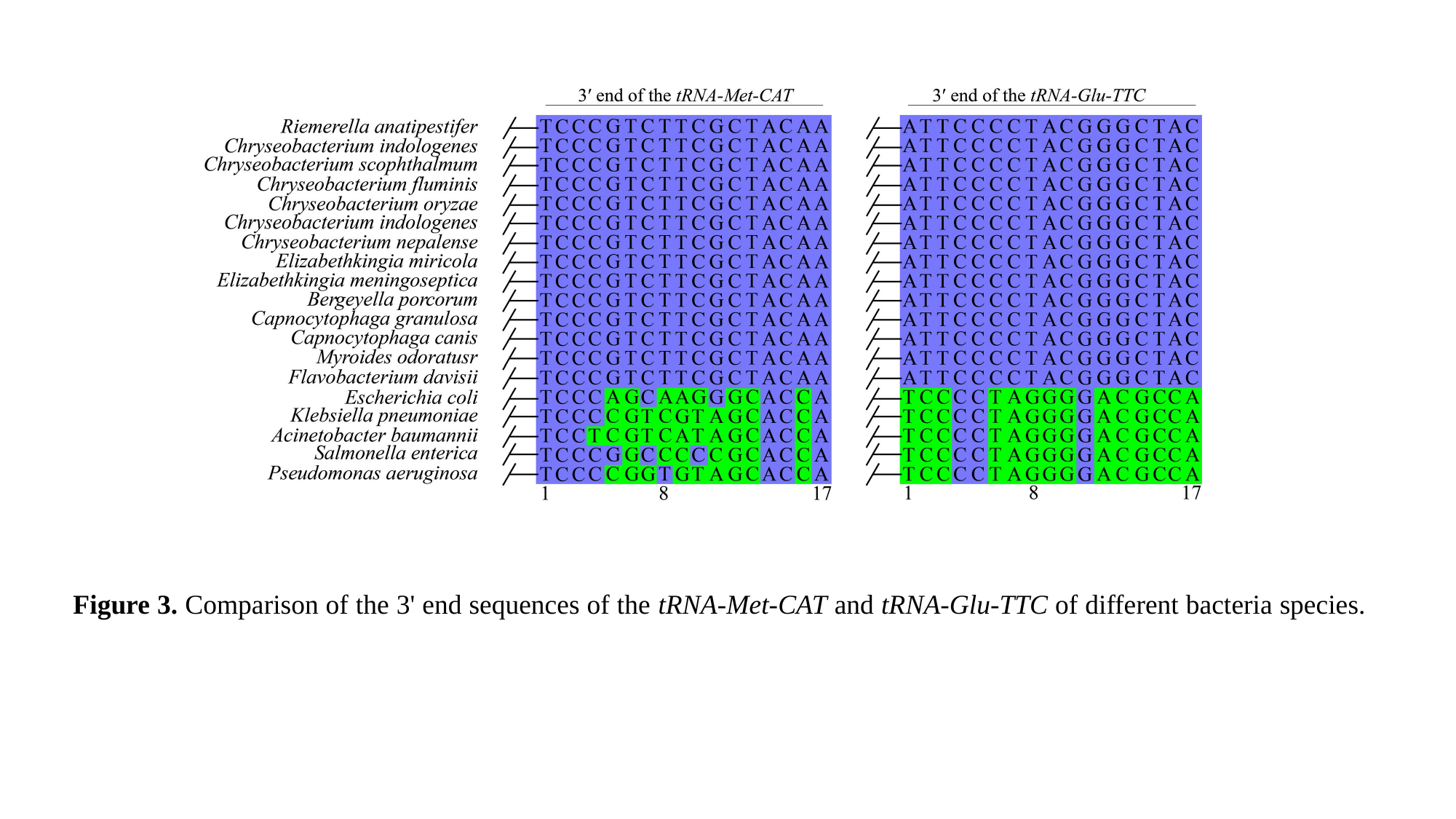

| |
| --- |
Figure 3. Comparison of the 3' end sequences of the tRNA-Met-CAT and tRNA-Glu-TTC of different bacteria species.

## Slide 4
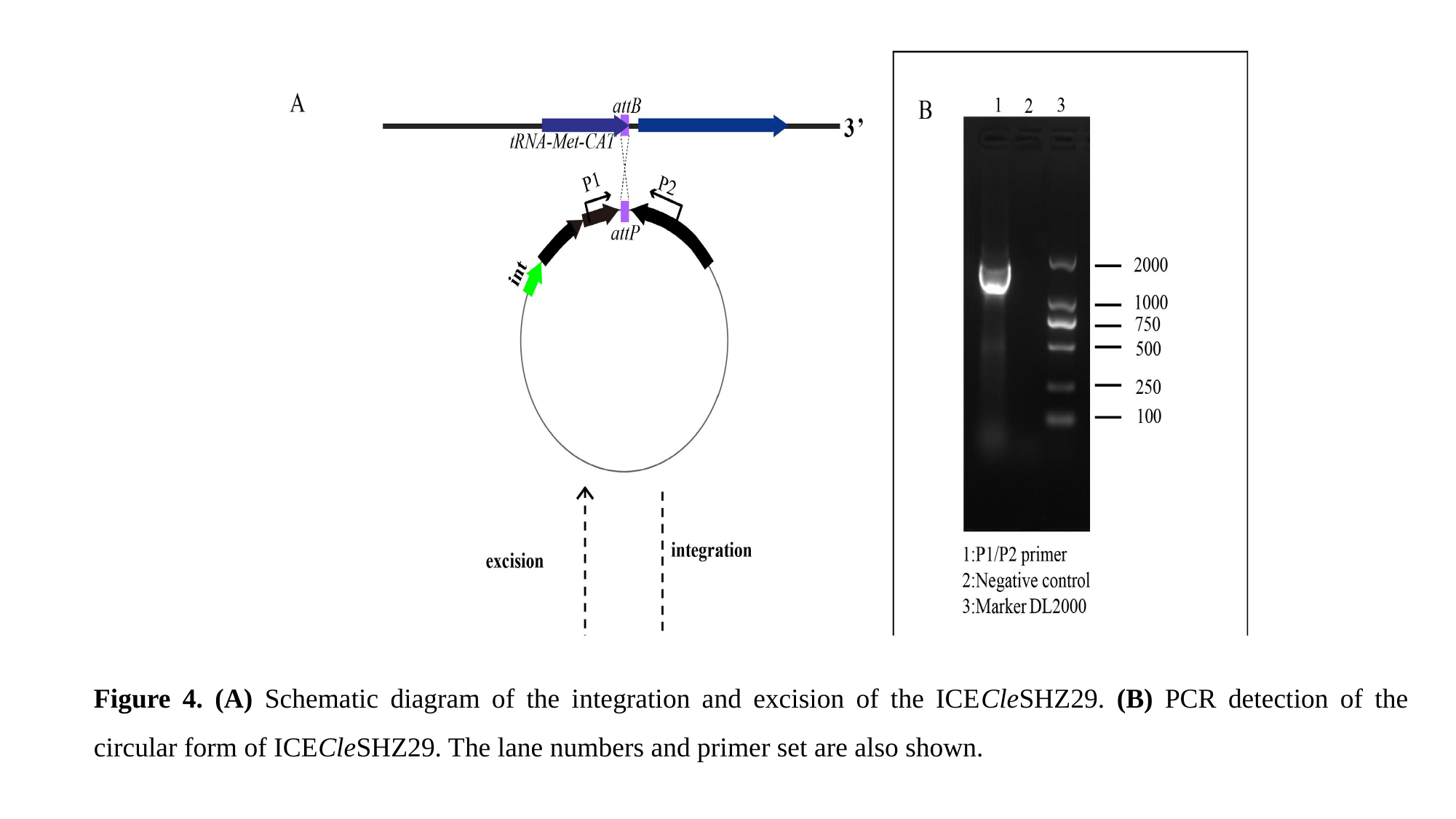

| |
| --- |
Figure 4. (A) Schematic diagram of the integration and excision of the ICECleSHZ29. (B) PCR detection of the circular form of ICECleSHZ29. The lane numbers and primer set are also shown.
