# Supplementary material for: ICECleSHZ29: Novel Integrative and Conjugative Element (ICE)-Carrying Tigecycline Resistance Gene tet(X6) in Chryseobacterium lecithinasegens
Source: Antibiotics (Basel). 2025 Oct 10;14(10):1002. doi: 10.3390/antibiotics14101002 (PMC12561888; doi:10.3390/antibiotics14101002)
Supplement: Supplementary file 1 [file antibiotics-14-01002-s001.zip › Supplementary figures/Supplementary Figure legends.docx]

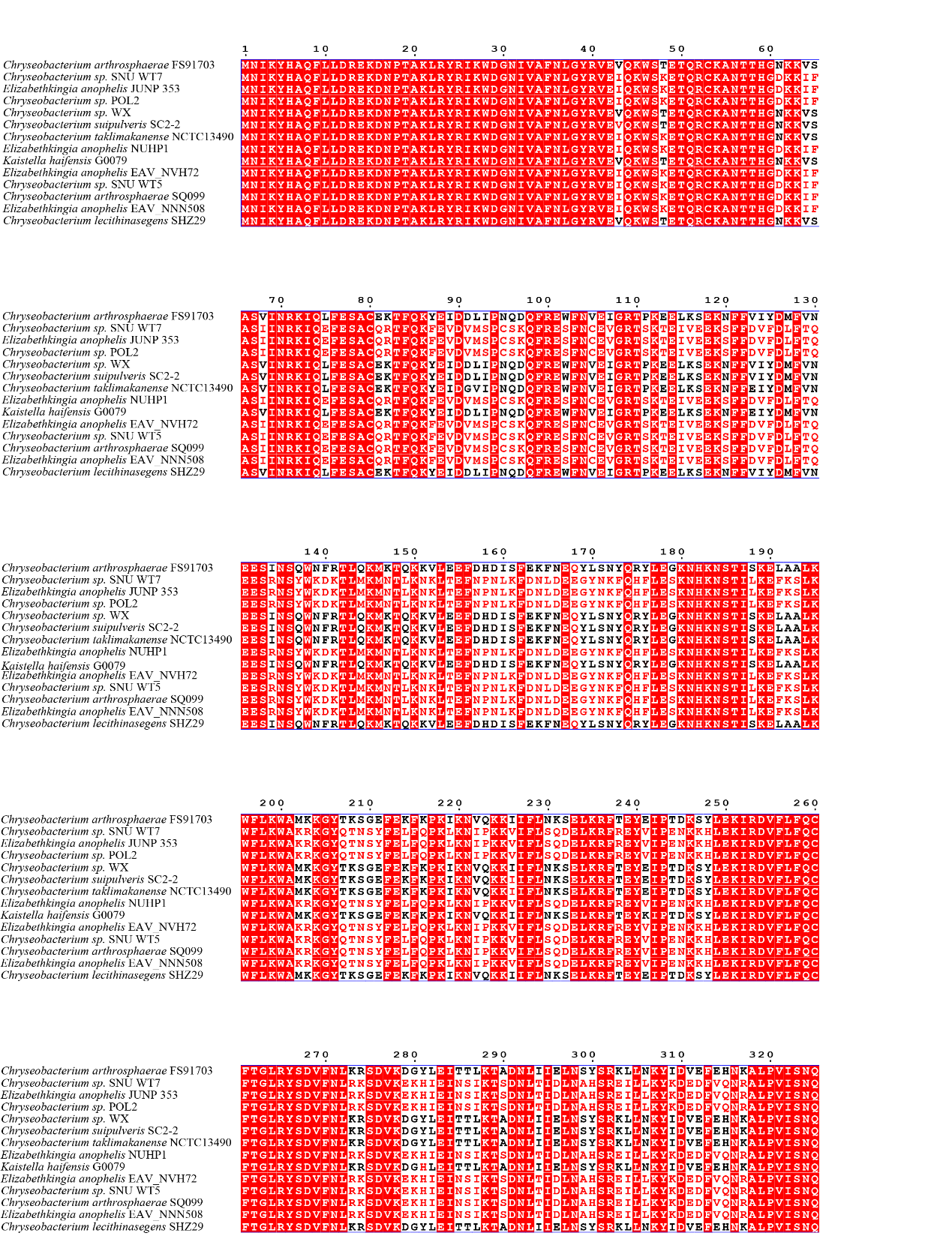


**Supplementary Figure 1.** BLAST alignment of integrase amino acid sequences from ICECleSHZ29-like integrative conjugative elements.

**
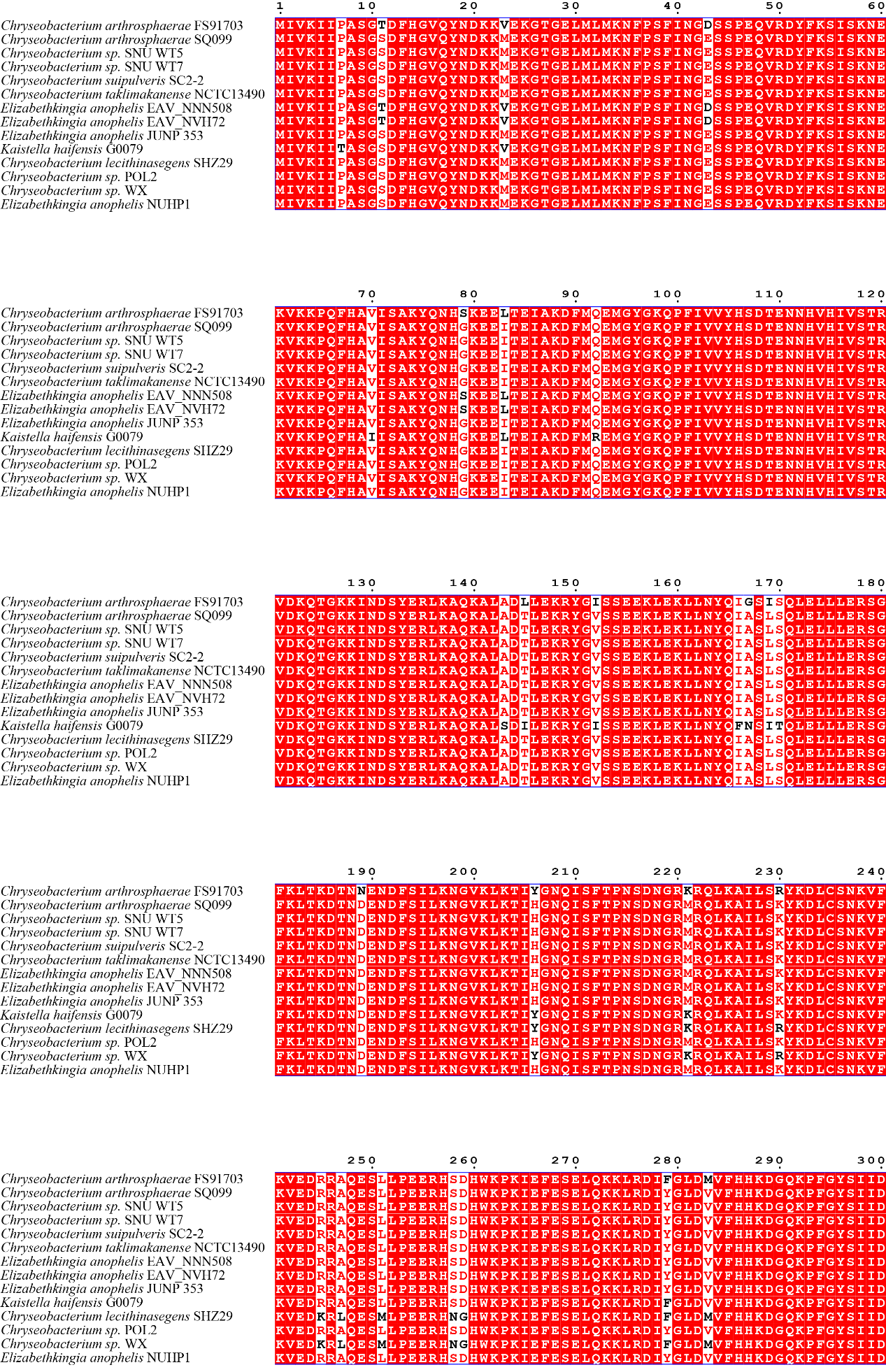
**

**Supplementary Figure 2.** BLAST alignment of relaxases amino acid sequences from ICECleSHZ29-like integrative conjugative elements.
