# Supplementary material for: ICECleSHZ29: Novel Integrative and Conjugative Element (ICE)-Carrying Tigecycline Resistance Gene tet(X6) in Chryseobacterium lecithinasegens
Source: Antibiotics (Basel). 2025 Oct 10;14(10):1002. doi: 10.3390/antibiotics14101002 (PMC12561888; doi:10.3390/antibiotics14101002)
Supplement: Supplementary file 1 [file antibiotics-14-01002-s001.zip › Supplementary Table S1.pdf]

Supplementary Table S1. Primers used for PCR Assay

Primer Name	Primer Sequence（5'-3'）	Program parameters	PCR reaction system	Purpose
P1	GTGGGAACAGAAAGCGAA	(95 °C, 5 min)+[(95 °C, 30 s)+(55 °C, 30 s)+(72 °C, 2 mins)]×35+(72 °C, 7 min) +(4 °C,∞)	P1/P2 (10 μmol/L): 1.5 μL each<br>2 ×pre Mix: 25μL<br>Template DNA: 2 μL<br>ddH_2O: up to 50 μL	For circular form detection
P2	CTACAAACCGGAAGAAGTCG			
P14	TGGGGAGGTTTAGGAATTGT	(95 °C,5 min)+[(95 °C, 30 s)+(55 °C, 30 s)+(72 °C, 10 min)]×35+(72 °C, 7 min) +(4 °C,∞)	P14/P16 (10 μmol/L): 1.5 μL each<br>2 ×pre Mix: 25μL<br>Template DNA: 2 μL<br>ddH_2O: up to 50 μL	To obtain the complete sequence of ICECleSHZ29 by gap-filling contigs 14 and 16 from our whole-genome sequencing assembly.
P16	TACTGCTCTTATGTCTCGGT			
