# Supplementary figures and images for: ICECleSHZ29: Novel Integrative and Conjugative Element (ICE)-Carrying Tigecycline Resistance Gene tet(X6) in Chryseobacterium lecithinasegens
Source: Antibiotics (Basel). 2025 Oct 10;14(10):1002. doi: 10.3390/antibiotics14101002 (PMC12561888; doi:10.3390/antibiotics14101002)

A

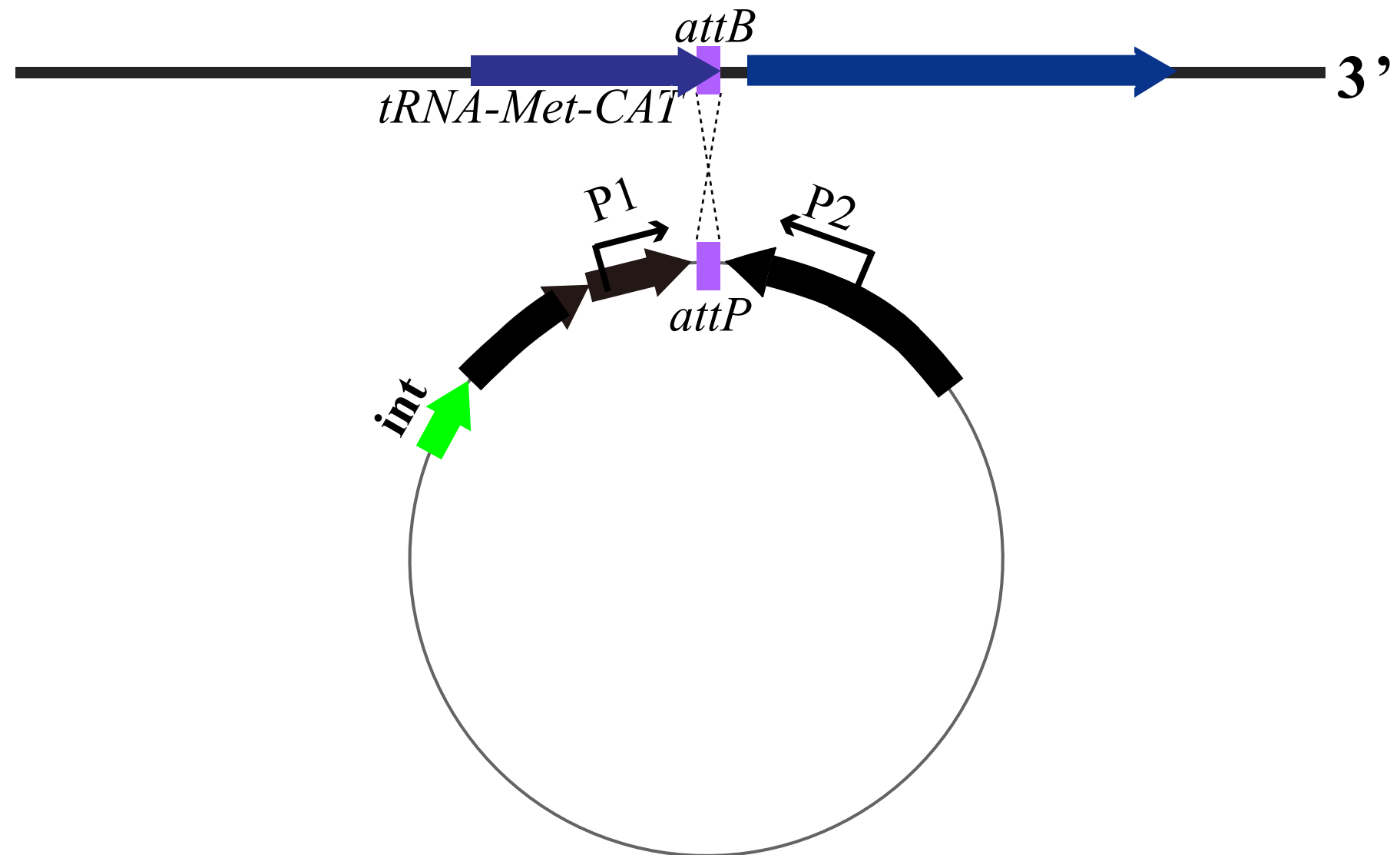

excision

integration

B

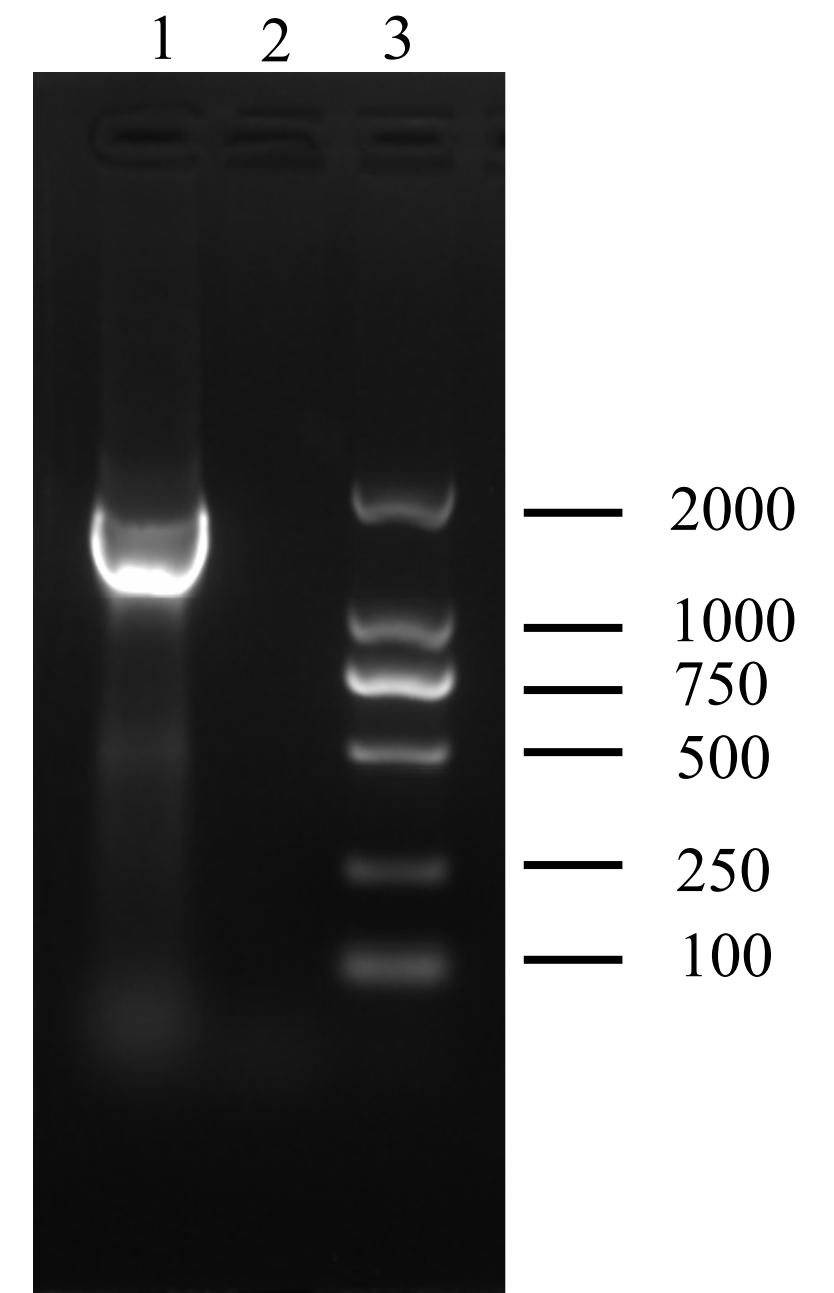

1:P1/P2 primer

2:Negative control

3:Marker DL2000

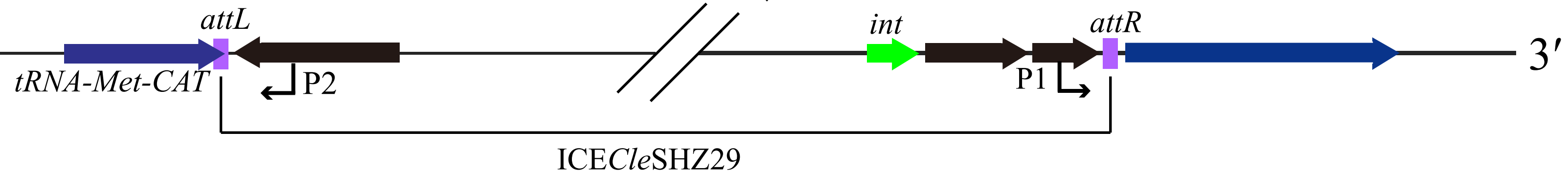

Supplement: Supplementary file 1 [file antibiotics-14-01002-s001.zip › Figures in PDF format/Figure 4.pdf]

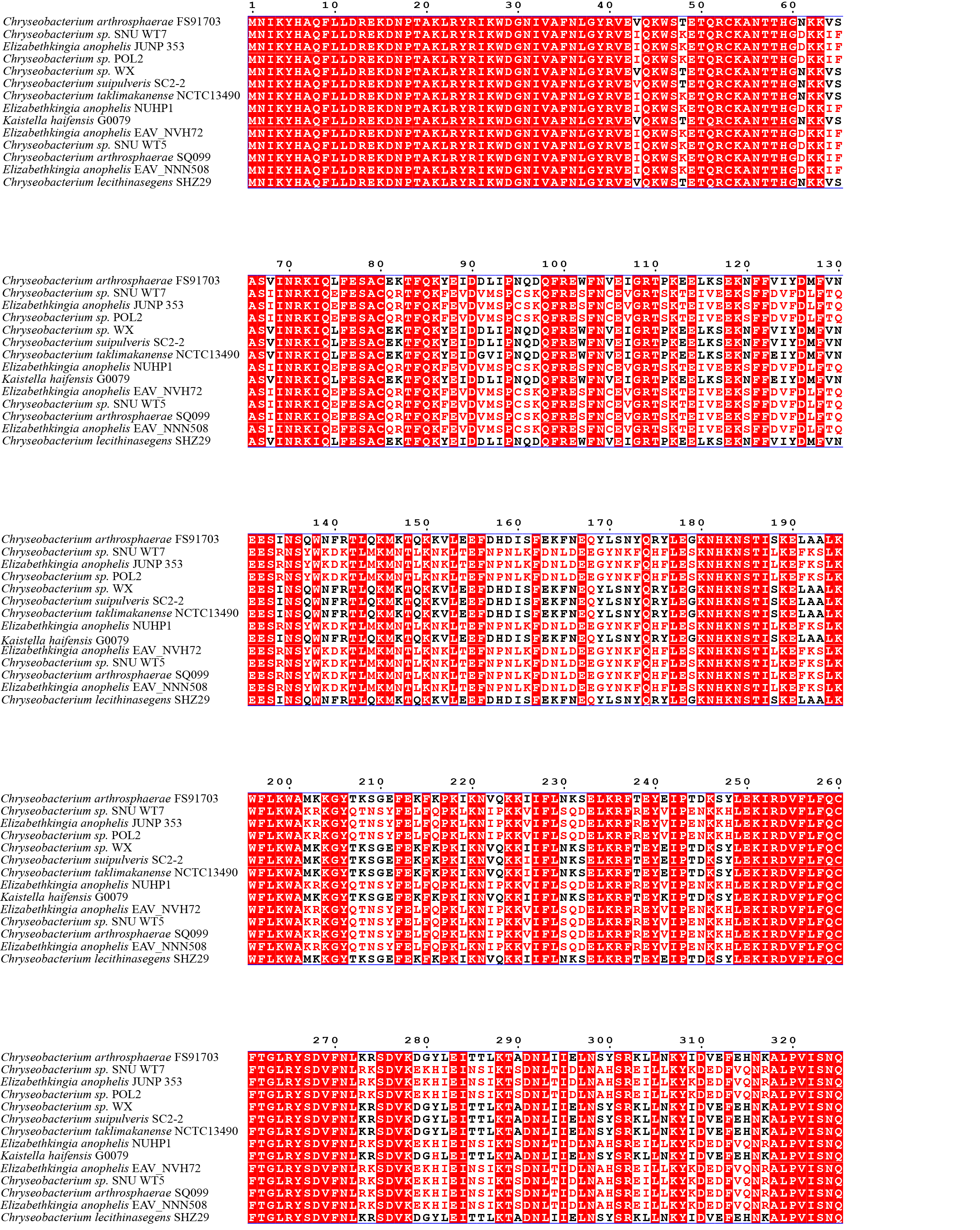

Supplement: Supplementary file 1 [file antibiotics-14-01002-s001.zip › Supplementary figures/Supplementary Figure. 1.tif]

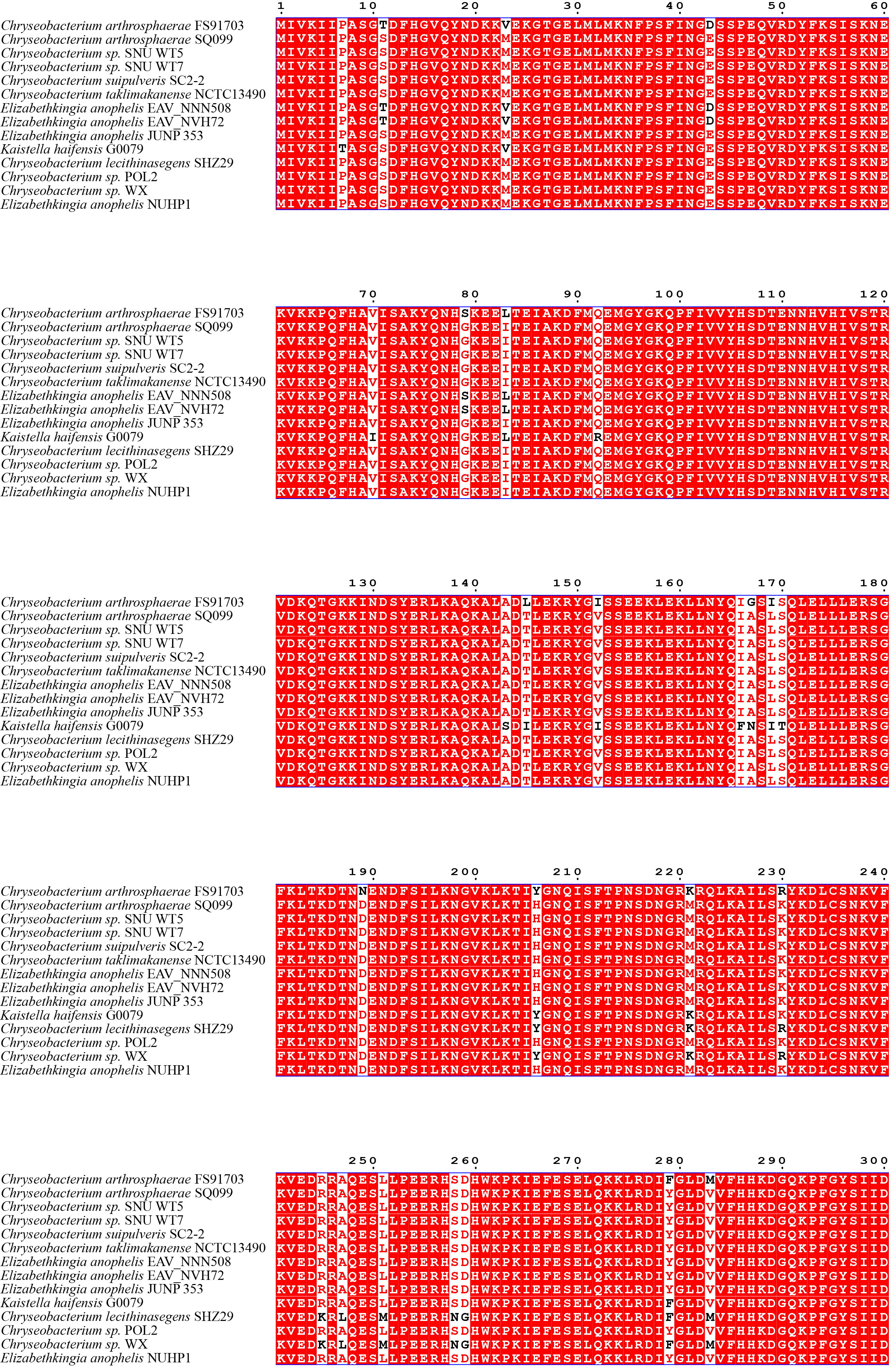

Supplement: Supplementary file 1 [file antibiotics-14-01002-s001.zip › Supplementary figures/Supplementary Figure. 2.tif]
