# Supplementary material for: ICECleSHZ29: Novel Integrative and Conjugative Element (ICE)-Carrying Tigecycline Resistance Gene tet(X6) in Chryseobacterium lecithinasegens
Source: Antibiotics (Basel). 2025 Oct 10;14(10):1002. doi: 10.3390/antibiotics14101002 (PMC12561888; doi:10.3390/antibiotics14101002)
Supplement: Supplementary file 1 [file Figure 1.pdf › Figures in PDF format/Figure 1.pdf]

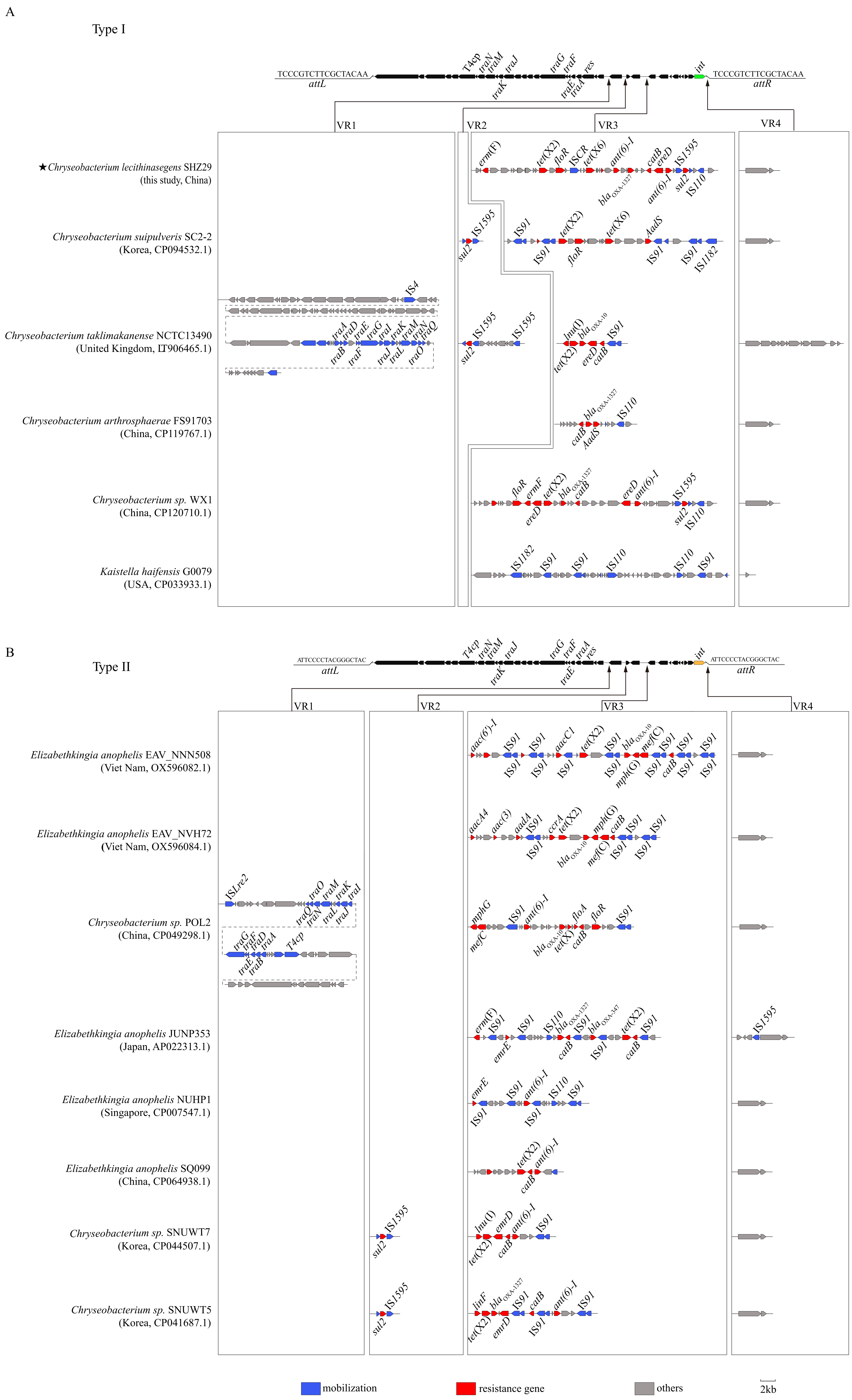
A
Type I
TCCCGTCTTCGCTACAA
attL
T4cp
traN
traM
traJ
traK
traG
traF
traE
traA
res
int
TCCCGTCTTCGCTACAA
attR
VR1
VR2
VR3
VR4
★Chryseobacterium lecithinasegens SHZ29
(this study, China)
Chryseobacterium suipulveris SC2-2
(Korea, CP094532.1)
Chryseobacterium taklimakanense NCTC13490
(United Kingdom, LT906465.1)
Chryseobacterium arthrosphaerae FS91703
(China, CP119767.1)
Chryseobacterium sp. WX1
(China, CP120710.1)
Kaistella haifensis G0079
(USA, CP033933.1)
B
Type II
ATTCCCCTACGGGCTAC
attL
ATTCCCCTACGGGCTAC
attR
Elizabethkingia anophelis EAV_NNN508
(Viet Nam, OX596082.1)
Elizabethkingia anophelis EAV_NVH72
(Viet Nam, OX596084.1)
Chryseobacterium sp. POL2
(China, CP049298.1)
Elizabethkingia anophelis JUNP353
(Japan, AP022313.1)
Elizabethkingia anophelis NUHP1
(Singapore, CP007547.1)
Elizabethkingia anophelis SQ099
(China, CP064938.1)
Chryseobacterium sp. SNUWT7
(Korea, CP044507.1)
Chryseobacterium sp. SNUWT5
(Korea, CP041687.1)
mobilization
resistance gene
others
2kb
